# Are We Nuts Over Coconuts? Studying the Effects of Coconut Oil on Low-Density Lipoprotein and Cardiovascular Diseases: A Systematic Review

**DOI:** 10.7759/cureus.24212

**Published:** 2022-04-17

**Authors:** Supriya Sekhar, Surabhi Makaram Ravinarayan, Ann Kashmer D.Yu, Fatma Kilic, Raghav Dhawan, Rubani Sidhu, Shahd E Elazrag, Manaal Bijoora, Lubna Mohammed

**Affiliations:** 1 Internal Medicine, California Institute of Behavioral Neurosciences & Psychology, Fairfield, USA; 2 Paediatrics, California Institute of Behavioral Neurosciences & Psychology, Fairfield, USA; 3 Plastic and Reconstructive Surgery, California Institute of Behavioral Neurosciences & Psychology, Fairfield, USA; 4 Anesthesiology, California Institute of Behavioral Neurosciences & Psychology, Fairfield, USA; 5 Psychiatry, California Institute of Behavioral Neurosciences & Psychology, Fairfield, USA; 6 Research and Development, California Institute of Behavioral Neurosciences & Psychology, Fairfield, USA

**Keywords:** myocardial infarction, coronary artery disease, cardiovascular diseases, low-density lipoprotein, bad cholesterol, lipids, plant oils, cocos nucifera, coconut oil

## Abstract

Coconut oil has been gaining popularity recently, especially with health enthusiasts claiming it to be the best fat for consumption. What is the ideal cooking fat? The answer that we are all looking for is just not solely based on one health consequence but several. Our study focuses on the cardiovascular aspects of using coconut oil by its influence on low-density lipoprotein (LDL). Cardiovascular diseases (CVDs) are the major cause of death and mortality worldwide. Hence, they are the focus of this study. For centuries, coconut oil has been used by several populations worldwide who consume it as part of their staple diets. However, they have also been consuming the flesh/meat of coconuts and decreased processed foods. One such population is the pacific islanders, who had increased LDL and decreased high-density lipoprotein (HDL) when they moved out of their natural habitat and accepted a more westernized diet. Even though coconut oil has a stronghold on the LDL aspect of the lipid parameters, which is our study's focus, it also increases HDL, whose effects on cardiovascular health are still controversial although it is called "good cholesterol."

Cardiologists now utilize the ratio of total to HDL cholesterol to assess CVD risk more reliably. There have not been many human studies to support coconut oil's LDL and CVD advantages, considering all these variables. A thorough search of five databases, including PubMed, PubMed Central, Google Scholar, Cochrane Library, and ScienceDirect, was done. The last search was done on October 8th, 2021. Studies were selected based on the following criteria: last five years, English language, human studies, randomized controlled trials (RCTs), systematic reviews and meta-analysis, narrative reviews, and cross-sectional studies were included using medical subject headings (MeSH) search and keyword search. Eight hundred and ninety-nine articles were found, and eight papers were picked after quality appraisal. These included one narrative review, three RCTs, one cross-sectional study, and three systematic reviews and meta-analyses. The results showed that coconut oil did not behave differently than other saturated fats to reduce LDL. One study showed that coconut oil did not increase LDL compared to additional saturated fat like butter or lard. Coconut oil also has antioxidant properties that may prevent oxidative stress that affects cardiovascular health. However, studies in this sector are limited.

## Introduction and background

Cardiovascular diseases (CVDs) are the leading cause of death globally. Around 17.9 million people died from CVDs in 2019, representing 32% of all deaths globally, of which 85% were due to heart attack [1]. These have a high incidence in developing countries. They are the cause of a significant percentage of fatalities in the middle-aged population in the United States [2]. A significant number of these deaths is coronary artery disease (CAD), caused by atherosclerotic disease that stems from high lipid levels [3]. A rise in lipid levels in our blood has been known to be caused by our diet and dietary ingestion of oils and fats [4]. One of the culprits is saturated fats, whose replacement with unsaturated fats can decrease the risk of developing atherosclerosis [3,4]. Hyperlipidemia is one of the most significant modifiable factors in CVD settings [5]. One of the first risk factors for atherosclerotic disease is high low-density lipoprotein (LDL) levels [3,6]. Management of LDL levels remains the main objective of dyslipidemia. LDL is composed of atherogenic proteins called lipoproteins in addition to VLDL and chylomicrons [6]. The LDL then infiltrates into the arterial wall causing atherosclerosis [6]. Coconut oil has been commonly consumed for several years by humans and is an integral part of food [7]. Other standard terms for this oil are tropical oil, lauric oil, or plant oil [7].

Coconut oil has 90% of saturated fat and is hence thought to have an increased effect on serum cholesterol levels [4,8]. However, a recent study suggests that coconut oil may benefit the body in a variety of ways [9]. It also has around 62% monounsaturated fatty acids (MUFAs), which has increased its popularity among consumers due to its effects on health [4,9]. With the increasing popularity of functional medicine, coconut oil usage, especially the virgin and extra virgin varieties, has gained popularity [9,10]. Lauric acid, a component of coconut oil, has a distinct impact on health than other saturated fatty acids (SFAs) such as palmitic acid, which is mostly found in butter, animal fat, and palm oil [10].

There have been reports of several advantages of consuming coconut oil on other entities of the serum lipids such as raising high-density lipoprotein (HDL) cholesterol which is termed "the good cholesterol" [8,9]. Apart from effects on the lipids, it is also known to have antimicrobial, anticancer, and anti-inflammatory effects and helps with weight loss, Alzheimer's, and atheroprotective functions. Coconut oil is commonly used in many tropical countries and has gained much fame in recent years despite its high percentage of saturated fats [4,8]. Even though specific guidelines have stated that intake of dietary saturated fat consumption needs to be minimized, there is no evidence that certain oils with saturated fats that are predominantly consumed in tropical countries have a negative effect [4].

The impact of coconut oil on LDL and its direct consequences on CVDs will be discussed in this study through a systematic review.

## Review

Material and methods

Guidelines

**Figure 1:**
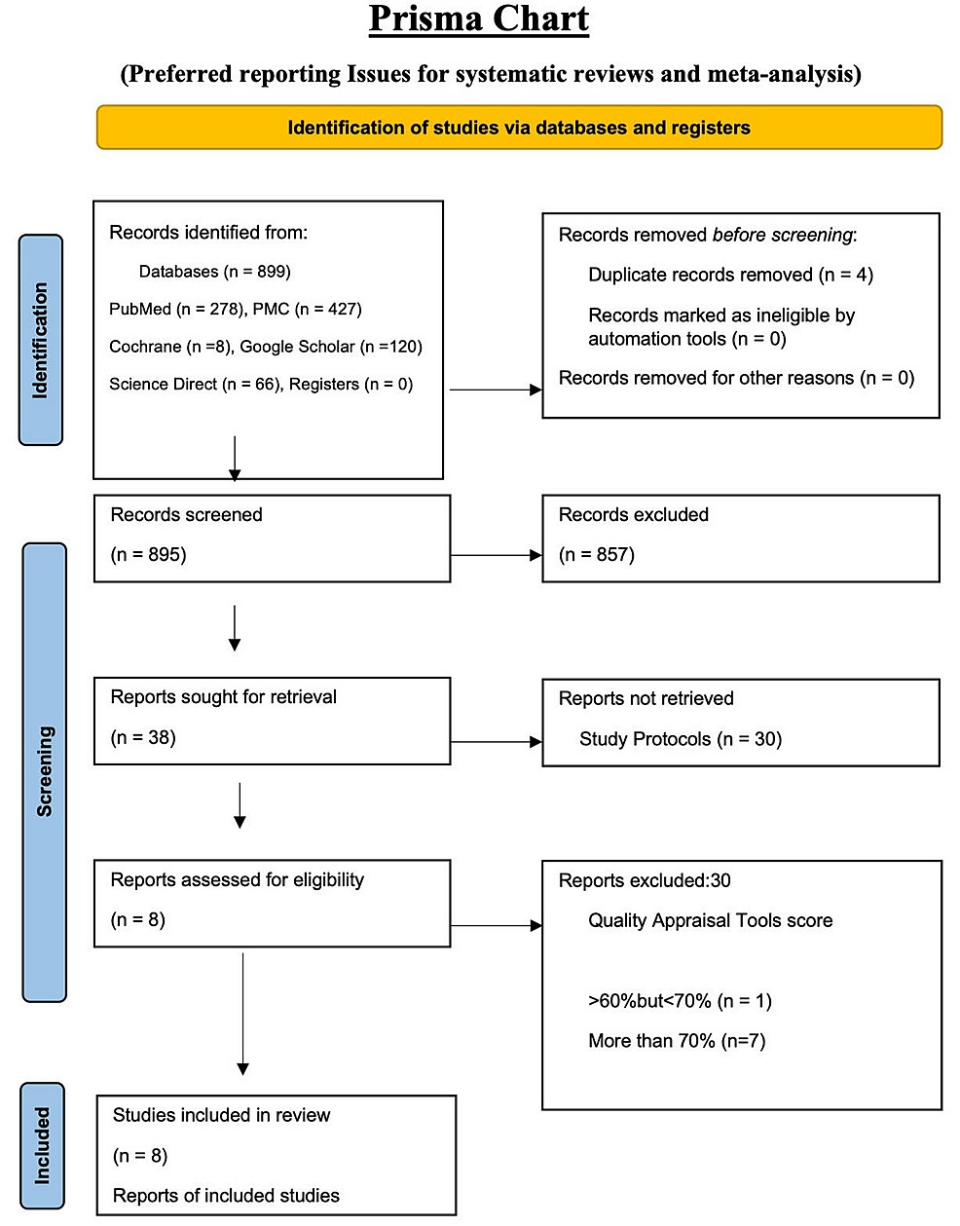
Study search and selection procedure flowchart PMC, PubMed Central.

The basic design of this systematic review was done according to 2020 PRISMA (Preferred Reporting Items for Systematic Reviews and Meta-Analyses) guidelines [4], as shown in Figure 1.

Review Question and PICO Strategy

Does coconut oil affect LDL, and if it does, does it affect CVDs?

Population (P): human; intervention (I): coconut oil ingestion; comparison (C): other oils if available; outcome (O): an effect on LDL and CVDs.

Eligibility Criteria

We did a thorough search of five databases. The databases used were PubMed, PubMed Central (PMC), Google Scholar, Cochrane Library, and ScienceDirect. Search keywords were: coconut oil, *Cocos nucifera*, plant oils, LDL, lipids, bad cholesterol, low-density lipoprotein, cardiovascular diseases, coronary artery disease, myocardial infarction (MI), and cardiac arrest. Medical subject headings (MeSH) keywords were identified and used along with Boolean operators like "OR" and "AND" for PubMed and other databases. After picking filters like articles from 2016 to 2021, human studies, articles in English, and articles from the last five years in other databases, 899 studies were identified. All references were then transferred to Excel (Microsoft, USA), and duplicates were removed. Four duplicates were identified and removed. After the initial abstract title screening, 38 articles were identified. After reviewing each article individually, eight articles - one narrative review, three randomized controlled trials (RCTs), one cross-sectional study, three systematic reviews, and meta-analyses - were finalized, after this study's quality appraisal. Figure 1 shows the PRISMA chart 2020 as a flow diagram showing the study selection process.

The MeSH Search Strategy

Coconut OR *Cocos nucifera* OR plant oil OR "coconut oil/administration and dosage" [Mesh] OR "coconut oil/adverse effects" [Mesh] OR "coconut oil/analysis" [Mesh] OR "coconut oil/chemistry" [Mesh] OR "coconut oil/metabolism" [Mesh] OR "coconut oil/pharmacology" [Mesh] OR "coconut oil/physiology" [Mesh] OR "coconut oil/therapeutic use" [Mesh] OR "coconut oil/toxicity" [Mesh] AND bad cholesterol OR lipids OR cholesterol OR low-density lipoprotein "cholesterol, LDL/adverse effects" [Mesh] OR "cholesterol, LDL/analysis" [Mesh] OR "cholesterol, LDL/biosynthesis" [Mesh] OR "cholesterol, LDL/blood" [Mesh] OR "cholesterol, LDL/chemistry" [Mesh] OR "cholesterol, LDL/classification" [Mesh] OR "cholesterol, LDL/drug effects" [Mesh] OR "cholesterol, LDL/genetics" [Mesh] OR "cholesterol, LDL/metabolism" [Mesh] OR "cholesterol, LDL/pharmacokinetics" [Mesh] OR "cholesterol, LDL/pharmacology" [Mesh] OR "cholesterol, LDL/physiology"[Mesh] OR "cholesterol, LDL/toxicity" [Mesh] OR "cholesterol, LDL/ultrastructure" [Mesh] AND CAD OR coronary artery disease OR MI OR myocardial infarction OR heart attack OR angina pectoris OR chest pain OR coronary thrombosis OR cardiac arrest OR "cardiovascular diseases/abnormalities" [Mesh] OR "cardiovascular diseases/classification" [Mesh] OR "cardiovascular diseases/diagnosis" [Mesh] OR "cardiovascular diseases/diet therapy" [Mesh] OR "cardiovascular diseases/drug effects" [Mesh] OR "cardiovascular diseases/drug therapy" [Mesh] OR "cardiovascular diseases/epidemiology" [Mesh] OR "cardiovascular diseases/ethnology" [Mesh] OR "cardiovascular diseases/etiology"[Mesh] OR "cardiovascular diseases/metabolism" [Mesh] OR "cardiovascular diseases/mortality" [Mesh] OR "cardiovascular diseases/physiology" [Mesh] OR "cardiovascular diseases/prevention and control" [Mesh] OR "cardiovascular diseases/statistics and numerical data" [Mesh] OR "cardiovascular diseases/therapy" [Mesh].

Inclusion/Exclusion Criteria

The literature search ended with 899 articles.

Inclusion: criteria involved were articles from 2016 to 2021, in English literature, and human studies. There were no filters applied in terms of age, gender, or ethnic origin. Studies that compared coconut oils with other oils and those that showed the effects of coconut oil on other entities of the lipid profile were also included.

Exclusion: editorials, case reports, and articles that were not open access during the last stages of the final search were also excluded.

Data Extraction

The first author extracted the following data: searched databases for the keywords, used inclusion and exclusion criteria, narrowed down studies after duplicate removal, reviewed abstract titles, and reviewed each report and quality appraisal application. Again, the last day for the search was October 8th, 2021.

The second and third authors individually verified abstract titles and the application of quality appraisal tools. Any disagreements were resolved by consensus and re-examination of the research in the issue.

Aim

To study whether coconut oil indeed had an effect on LDL and whether it affected CVDs.

Quality Assessment of the Studies

All studies had a quality appraisal done.

Here, we use the Cochrane risk bias assessment tool for RCTs, and each of the three studies was reviewed on seven criteria to look for biases. Scores were reported as either high quality, low quality, or unclear nature. We used 60% as the cut-off. Two out of the three RCTs scored >70. One study scored >60% but <70%. We also used A MeaSurement Tool to Assess systematic Reviews (AMSTAR) for systematic reviews, New Castle Ottawa for cross-sectional study, and the Scale for the Assessment of Narrative Review Articles (SANRA) for narrative reviews, as can be seen in Table 1.

**Table 1:** Quality assessment of studies AMSTAR 2, Assessment of Multiple Systematic Reviews 2, Cochrane Risk of Bias Assessment Tool; NOS, Newcastle Ottawa Scale; SANRA 2, Scale for the Assessment of Narrative Review Articles 2; SR & MA, Systematic Review and Meta-Analysis; RCT, randomized controlled trial; NR, narrative review; CSS, cross-sectional study.

Study type	Quality appraisal tool	Total score	Study features	Accepted score (>70%)	Accepted studies
AMSTAR 2	SR & MA	16	Sixteen items: 1. Did the research questions and inclusion criteria for the review include the components of PICO? 2. Did the report of the review contain an explicit statement that the review methods were established before the conduct of the review and did the report justify any significant deviations from the protocol? 3. Did the review authors explain their selection of the study designs for inclusion in the review? 4. Did the review authors use a comprehensive literature search strategy? 5. Did the review authors perform study selection in duplicate? 6. Did the review authors perform data extraction in duplicate? 7. Did the review authors provide a list of excluded studies and justify the exclusions? 8. Did the review authors describe the included studies in adequate detail? 9. Did the review authors use a satisfactory technique for assessing the risk of bias (RoB) in individual studies that were included in the review? 10. Did the review authors report on the sources of funding for the studies included in the review? 11. If meta-analysis was justified, did the review authors use appropriate methods for statistical combination of results? 12. If a meta-analysis was performed, did the review authors assess the potential impact of RoB in individual studies on the results of the meta-analysis or other evidence synthesis? 13. Did the review authors account for RoB in individual studies when interpreting/discussing the results of the review? 14. Did the review authors provide a satisfactory explanation for, and discussion of, any heterogeneity observed in the results of the review? 15. If they performed quantitative synthesis, did the review authors carry out an adequate investigation of publication bias (small study bias) and discuss its likely impact on the results of the review? 16. Did the review authors report any potential sources of conflict of interest, including any funding they received for conducting the review? Scored as YES or NO. Partial Yes was considered a point	10	Study [4], [5], [8]
SANRA 2	NR	12	Six items: justification of the article’s importance to the readership, statement of concrete aims or formulation of questions, description of the literature search, referencing, scientific reason, and appropriate presentation of data. Scored as 0, 1, or 2	9	Study [7]
New Castle and Ottawa	CSS	10	Selection: (maximum three stars). 1. Representativeness of the sample: (a) Truly representative of the average in the target population (all subjects or random sampling). (b) Somewhat representative of the average in the target population (non-random sampling). (c) Selected group of users. (d) No description of the sampling strategy. 2. Non-respondents: (a) Comparability between respondents' and non-respondents’ characteristics is established, and the response rate is satisfactory. (b) The response rate is unsatisfactory, or the comparability between respondents and non-respondents is unsatisfactory. (c) No description of the response rate or the characteristics of the responders and the non-responders. 3. Ascertainment of the exposure (risk factor): (a) Validated measurement tool. (b) Non-validated measurement tool, but the tool is available or described. (c) No description of the measurement tool. Comparability: (maximum two stars). 1. The subjects in different outcome groups are comparable, based on the study design or analysis. Confounding factors are controlled. (a) The study controls for the most important factor (select one). (b) The study control for any additional factor. Outcome: (maximum two stars). 1. Assessment of the outcome: (a) Independent blind assessment. (b) Record linkage. (c) Self-report. (d) No description. 2. Statistical test: (a) The statistical test used to analyze the data is clearly described and appropriate, and the measurement of the association is presented, including confidence intervals and the probability level (*p*-value). (b) The statistical test is not appropriate, not described or incomplete	8	Study [9]
Cochrane Risk of Bias Assessment Tool	RCT	7	Seven items: random sequence generation and allocation concealment (selection bias), selective outcome reporting (reporting bias), other sources of bias, blinding of participants and personnel (performance bias), blinding of outcome assessment (detection bias), and incomplete outcome data (attrition bias). Bias is assessed as low risk, high risk, or unclear	5	Study [3], [10], [11]

Results

Search Outcome

After the extensive search, 899 records were identified from thoroughly searching five databases. All records were transferred to Microsoft Excel, and four duplicates were removed. The records were initially reviewed based on the titles and abstracts. After applying the inclusion/exclusion criteria, a total of 857 were excluded, including editorials, observational studies, studies published before January 2016, non-English reports, and non-human studies. Thus, a total of 895 studies were screened. Thirty were excluded for reasons after careful screening of the abstracts, results, and safety profiles. As full-text articles were unavailable, four were excluded in the final stages among the 30 articles, and eight relevant studies were selected for review after the quality assessment. This systematic review was not registered. Below is a summary of the final studies included. Coconut oil was compared to other tropical oils, butter and lard, and their effects on LDL and CVDs, as can be seen in Table 2.

**Table 2:** Summary of the studies included NR, narrative review; TC, triglyceride; HDL, high-density lipoprotein; LDL, low-density lipoprotein; USPO, unsaturated plant oils; SFs, saturated fats; CVDs, cardiovascular diseases; RCT, randomized controlled trial; CSS, cross-sectional study; SR & MA, systematic review and meta-analysis; EVCO, extra virgin coconut oil; MUFA, monounsaturated fatty acid; PUFA, polyunsaturated fatty acid.

Year of study	Design	Participants, *n*	Comparator oil/fat	Results	Funding	Declaration of interest
Eyres et al. 2016 [7]	NR	181	Butter, soybean oil, safflower oil, palm oil, corn oil, extra virgin olive oil	Coconut oil increased TC, HDL, and LDL when compared to USPO but not as much as butter did. This suggests that coconut oil vs. other SFs were not that different on lipids. Does not support that coconut oil was a healthy oil concerning CVDs	Yes	None
Vijayakumar et al. 2016 [3]	RCT	200	Sunflower oil	There was not much change in those who consumed coconut oil vs. sunflower oil concerning cardiovascular risk due to its influence on lipid parameters	Yes	None
Khaw et al. 2018 [10]	RCT	96	Butter, olive oil	Coconut oil behaved similarly to olive oil on LDL and did not raise LDL as much as butter. Coconut oil raised HDL more than butter and olive oil. However, the primary endpoint was LDL, and the effect on CVDs was not studied. Furthermore, it recommends a reduction in saturated fat intake	Yes	None
Palazhy et al. 2018 [12]	CSS	153	Sunflower oil	No difference in the effects of coconut oil on LDL when compared to sunflower oil. Lower levels of vitamin C in the sunflower group than in coconut oil, causing more lipid peroxidation and less antioxidant properties	Yes	Unknown
Schwingshackl et al. 2018 [5]	SR & MA	2,065	Butter, lard safflower, sunflower, rapeseed, flaxseed, corn, olive, soybean, palm	Coconut oil safflower, sunflower, rapeseed, flaxseed, corn, olive, soybean, and palm decreased LDL more than butter and lard	Not mentioned	Not mentioned
Neelakantan et al. 2020 [8]	SR	418	Nontropical vegetable oils and palm oil	Coconut oil increases LDL more than palm oil. It increased LDL when compared to nontropical oils	Yes	None
Junior et al. 2021 [11]	RCT	51	EVCO compared to placebo	EVCO did not have an antihypertensive effect in patients with stage 1 hypertension. It also showed no effect on blood pressure differences and oxidative stress levels in humans. LDL levels were slightly elevated when compared to the placebo group	Yes	None
Unhapipatpong et al. 2021 [4]	SR	54	MUFA- and PUFA-rich oils	Substituting coconut oil with PUFA- and MUFA-rich oils significantly increased TC and HDL-c but not LDL-c, while coconut oil substituted for other SFs significantly increased HDL-c. Moreover, the replacement of butter with coconut oil significantly decreased LDL-c and TC and increased HDL-c	Yes	None

Discussion

The big question - What is the best fat or oil to use in cooking to avoid the onset of CVDs. Does something like that exist? This study was intended to evaluate the association of one such oil that is commonly used by many populations - coconut oil usage and its effects on LDL and CVDs. Although only a few studies have been done about coconut oil and its effect on LDL, its effects remain questionable. The study also did not show a good amount of research done on humans to base conclusions on the benefits or effects of the oil in question on LDL and eventually on cardiovascular health [7].

Epidemiology

Several populations globally consume coconut products as a part of their regular daily diet and as a primary cooking medium [4,8]. These include and are not limited to people mainly in Asia - India, the Philippines, Sri Lanka, and the Polynesian Islands. A previous literature review of eight studies argued that consumption/ingestion of coconut oil had had no deleterious effects on cardiovascular health [7]. Several tropical countries have consumed coconut oil for many decades. However, there is not enough research to prove that saturated fats from these oils cause CVDs. Thus, the topic is still up for debate. As many distinct factors impact a single health outcome or indicator simultaneously, observational studies and these demographic groups cannot prove causality and are prone to confounding [7].

Composition

**Figure 2:**
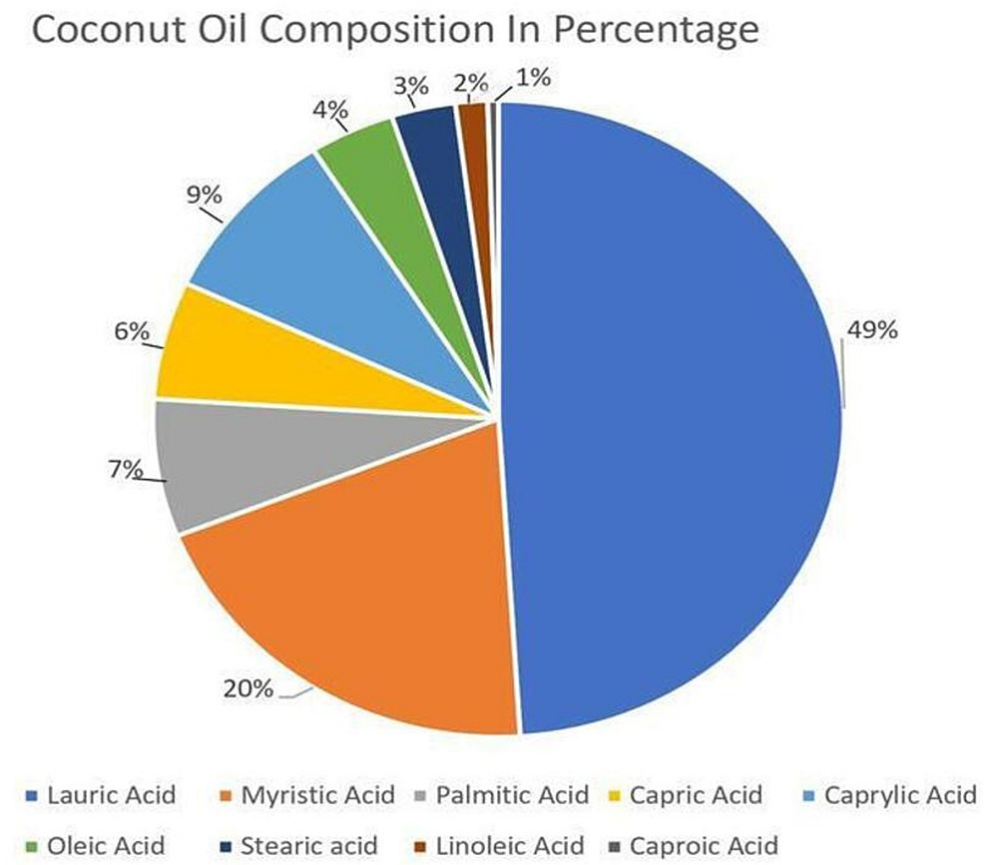
Coconut oil composition in percentage [15]

Coconut oil is 100% fat, of which 80-90% is saturated fat [13]. It also contains most of the medium-chain fatty acids (MCFAs) [14]. It solidifies at cold temperature, is soft at room temperature, and liquefies when warmed [14]. Coconut oil has different forms that are produced from the way they are processed. Hence, there possibly is a difference in the effects when a particular composition is used. There may be a vast difference between using extra virgin coconut oil (EVCO) versus a traditional variety of coconut oil. The phenolic compounds and associated antioxidant properties vary. The processing of oils can change the overall content, chemical structure, and eventual effects on the metabolism [10]. Coconut oil is also composed of: lauric acid - 49%, myristic acid - 20%, palmitic - 7%, capric - 6%, caprylic - 9%, oleic - 4%, steric - 3%, linoleic - 2%, and caproic acid - 1% [15], as can be seen in Figure 2.

Medium- and Long-Chain Fatty Acids and Their Absorption

**Figure 3:**
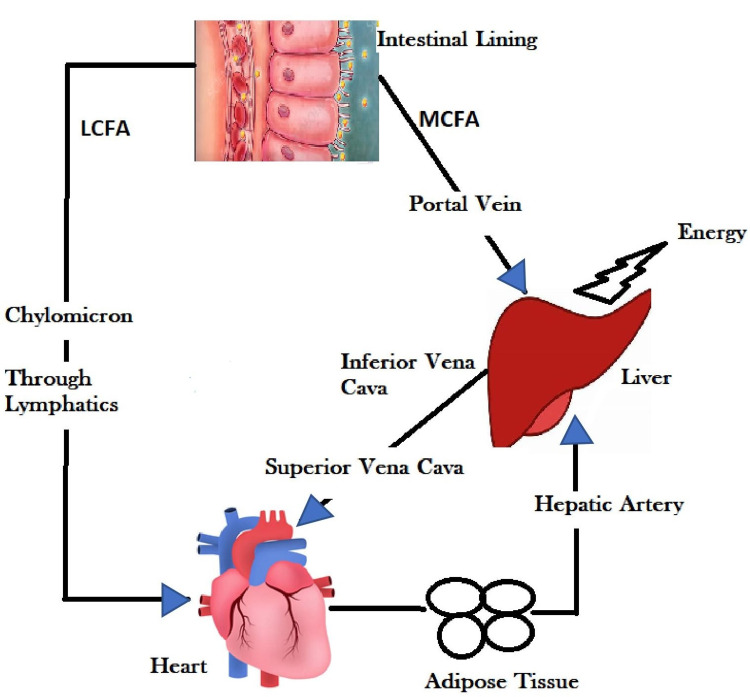
Metabolism of LCFAs and MCFAs LCFAs, long-chain fatty acids; MCFAs, medium-chain fatty acids. Figure credit: done by the original author from the description above.

Coconut oil is composed of MCFAs and long-chain fatty acids (LCFAs). As opposed to the LCFA, MCFA is directly absorbed by the intestinal lumen without the help of pancreatic lipases or biliary acids into the liver via the portal vein. Some are directed to form ketones and provide energy directly from the liver, while some recirculate to the systemic circulation via the inferior vena cava into the systemic circulation. They do not require pancreatic lipase and also do not require carnitine. Therefore, whatever is absorbed is mainly turned into energy, unlike LCFA that is stored in adipose tissues. LCFA requires chylomicrons that act as carriers in the lymphatic system after getting absorbed by the intestinal lumen and directly into the systemic circulation to get stored as fat [16]. They also do not depend on bile salts and acids for absorption. Thus, instead of LCFAs that enter the systemic circulation and are carried to adipose tissue and stored as fat, they are immediately accessible for energy [16], as can be seen in Figure 3.

The Global Coconut Oil Producer

Throughout history, coconut oil, both whole and extracted, has been a staple in the food supply of many nations in Asia and the Pacific Rim. However, other seed oils have recently begun to take the role of coconut oil. This is not very reassuring since the advantages of eating enough coconut oil are numerous [14]. As the world's leading producer of coconut oil, the Philippines has shown how lucrative this industry can be. The coconut industry directly or indirectly employs about one-third of the population in the Philippines [17].

Varieties

Depending on how the coconut meat is removed, there are five types of coconut oils: refined, bleached, and deodorized (RBD), virgin, and EVCO. RBD oil is made from dried coconut kernels or flesh produced by smoke drying, sun drying, or combining the two methods [17].

American Heart Association Recommendation

The American Heart Association (AHA) has emphasized the role of saturated fats in its recommendations over the years. Saturated fats such as coconut oil and other oils that are tropically derived should be replaced with unsaturated fatty acids, according to a scientific guideline statement that was issued by the AHA in 2017. Coconut oil was found to elevate dangerous LDL cholesterol levels in seven studies by the AHA. Since then, AHA recommended that coconut oil should therefore be avoided, and all saturated fat should be limited. They recommended that those at risk of already having heart disease consume no more than 6% of the total calories from saturated fat or roughly 13 g based on a 2000-calorie diet [18,19]. Also, limiting cholesterol intake to <300 mg/day was found to be beneficial [20].

The AHA warns consumers that the HDL improvements seen with diet or drug treatment cannot be directly linked to changes in CVD occurrences and therefore encourages Americans to look at the LDL changes as affected by various fats alone regarding their role in CVDs [20]. The National Lipid Association Expert Panel strongly recommends (Grade A evidence) a diet low in SFA (<7% of energy) [21]. One tablespoon of coconut oil has around 12 g of saturated fats, which is close to the maximum limit (replacement of saturated fat with dietary MUFAs). Moreover, polyunsaturated fatty acids (PUFAs) reduce ASCVD risk [22,23].

Antioxidant Effects - Influence on CVD Risk?

Human studies on the antioxidant properties of coconut oil are uncommon and inconsistent. The two most prevalent fatty acids in coconut oil are lauric acid and myristic acid [24]. They are MCFAs that are readily absorbed in the circulation, which might explain why coconut oil is broken down and oxidized by the liver to increase energy expenditure. When compared to other saturated fats, it may provide a variety of health benefits [7,24]. Dietary antioxidants and endogenous antioxidants effectively aid in the scavenging of free radicals produced excessively in the body. Vitamin C is a chain-breaking antioxidant that prevents the chain reactions of lipids but oxidation of LDL oxidation. Vitamin C levels in CAD patients are lower in studies on patients done in the past. Although some research suggests that vitamin C can help lower cholesterol, the cardioprotective benefits of the intake of vitamin C have yet to be proven clearly. One study revealed that vitamin C lowered LDL and CAD in the sunflower oil group compared to the coconut oil group, but a comparison could not be established due to a lack of compatible human research [9]. Other antioxidants present in coconut oil are vitamin E, phytosterols, and phenolic compounds. Furthermore, replacing coconut oil with PUFAs and MUFAs appears to lower coronary heart disease (CHD) incidence [9]. As a result, the current evidence does not support the use of coconut oil in the treatment and prevention of CVDs, and general SFA consumption recommendations (limited to 9% of total calorie intake) should be followed [25].

LDL and CVDs

A vast spectrum of research papers back up the LDL theory of atherosclerosis and CVDs [3]. In addition, there is evidence that high blood LDL cholesterol levels may contribute to atherosclerotic CVD in the absence of other risk factors [6].

Coconut Oil and LDL

According to a recent poll, 72% of the general population in the United States considers coconut oil to be a good fat compared to 37% of nutritionists. The popular press's promotion of coconut oil is to blame for the lay and professional opinion gap. Even though coconut oil increases LDL content, it also significantly increases HDL [9]. Coconut oil, however, does not make the liver produce cholesterol and hence by increasing HDL, it can decrease the LDL/HDL ratio [26]. Saturated fat consumption has been shown to elevate LDL-c levels through downregulating LDL receptors. However, the extent to which saturated fat consumption affects LDL-c may also be affected by other variables such as dietary cholesterol intake, variability in individual SFAs, and individual variations in response to diet [23].

*Coconut Oil on CVDs*

There has been no research or RCTs that have explicitly examined the effect of coconut oil on CVD mortality or outcomes [10]. Observational studies done in a prior study showed that Indigenous communities that eat coconut as their primary food had a lower risk of CVDs, although their diets do not contain large amounts of saturated fats. Furthermore, instead of coconut oil, these people ate coconut meat or squeezed coconut as a part of their typical diet [7]. Although some studies have indicated a reduction in total cholesterol when unprocessed coconut oil is added to an otherwise regular diet, blood cholesterol levels seldom change [12]. According to numerous studies, coconut oil has the opposite impact on serum cholesterol in those with low and high serum cholesterol. We observe a rise in serum total cholesterol, LDL cholesterol, and particularly HDL cholesterol in those with low serum cholesterol. Hypercholesterolemia, on the other hand, has a reduction in total and LDL cholesterol, as previously mentioned [14]. Coconut oil proponents claimed that those who ingested coconut oil have a low risk of CVDs. One such study mentioned that Tokelauans and Pukapukans get around 34% and 63% of their daily energy from coconut, respectively. Tokelauan individuals who moved to New Zealand had a greater level of total cholesterol. Despite having a reduced saturated fat intake, those who moved to New Zealand had higher total cholesterol, LDL, and decreased levels of HDL than those who stayed in Tokelau [8]. However, because of the observational and ecological characteristics of the investigations, these findings should be regarded with care with the significant risk of being confounded by the cultural/traditional diet, which contains lots of fish and less processed foods [8]. A study done by Eyers et al. refuted the widespread assertions that coconut oil was beneficial for lowering the risk of CVDs. In terms of its impact on the various components of the blood lipids, there was no indication that coconut oil behaved consistently different from other saturated fats. Although the evidence of a link between coconut oil consumption and CVD risk factors is generally of poor quality, it appears that when compared to unsaturated plant oils, coconut oil raised total cholesterol, HDL, and LDL, though not as much as butter does. The effect of coconut oil consumption on the total cholesterol to HDL ratio has not been widely reported. It asserts that substituting unsaturated fats for coconut oil does not lower the risk of CVDs. Francisco A. O. Júnior et al. added a focus on EVCO for supplementation on antihypertensive effects in patients with stage I hypertension. It stated that there were no effects on the blood pressure variability or levels of oxidative stress in humans. However, the limitation of the study was that a dose of 10 mL/day of coconut oil supplementation was used. Khaw et al. conducted an RCT that compared the effects of different types of oils, including coconut oil, olive oil, and butter, on LDL and CVD risk. One study showed a higher LDL level in butter than in coconut oil; however, had the same effect on LDL between coconut oil and olive oil. There was not a direct correlation between the effects of coconut oil on CVDs, but a 15% increase in the risk of CVDs occurs with every 1 millimolar increase in the LDL count. Therefore, it can be assumed that it directly negatively affects cardiovascular health. Vijayakumar et al. did an RCT where the effect of coconut oil and sunflower oil was studied, and it was found that there was not much change in the effects on LDL when compared between these two oils; however, the study included patients that were on statins which was a limitation. A systematic review done by Unhapipatpong et al. showed that coconut oil decreased LDL when compared to other SFAs, which was also not statistically significant. Thus, it is shown that coconut oil, a plant-derived saturated fat, had an inconclusive health impact because it increased good and bad cholesterol after consumption. In addition, coconut oil increased LDL compared to other PUFAs, although the effect was not statistically significant. Also, coconut oil was associated with a lower LDL than other SFAs, which was also not statistically significant.

Another systematic review and metanalysis by Nithya et al. showed that coconut oil consumption significantly increased total cholesterol, LDL cholesterol, and HDL concentrations compared with non-tropical vegetable oils. Since it increased both HDL and LDL, the efforts to reduce CVDs by just increasing the HDL component were not proven enough. Furthermore, it was suggested that the quantity of saturated fat in the diet be limited. Palazhy et al. conducted a cross-sectional study that compared subjects from two groups consuming coconut oil and sunflower oil separately. It was discovered that individuals who ate coconut oil did not show hypercholesterolemia compared to the sunflower oil groups. However, compared to those who consumed coconut oil, those who consumed sunflower oil showed higher levels of oxidative stress and lower amounts of vitamin C. Low levels of vitamin C are known to cause CVDs by increasing lipid peroxidation. Schwingshackl et al. did a systematic review and meta-analysis of several types of oils, including unsaturated fatty acids and coconut oil, which were more effective in reducing LDL than in SFA-rich foods like butter or lard. As a result, the existing limited data does not support the use of coconut oil to prevent or treat CVDs, and general recommendations on SFA intake (limited to 9% of total energy intake) should prevail [25]. There has been no documented clinical research comparing the direct effects of coconut oil and other dietary oils on CVDs, but it is advised adequately against using coconut oil as it raises LDL risk factor for CVDs and has no proven beneficial effects [12].

## Conclusions

Limitations and future prospective

There has not been single research that looked at the mortality effects of coconut oil intake on CVDs such as MI or cardiac arrest in individuals with high LDL. Studies comparing the use of different types of coconut oil have not been done. The physiological effects of coconut oil on the metabolic parameters can be influenced by the way it is processed, and hence their health effects can be different. Studies that compare a particular type of coconut oil in larger dosages have not been studied, and this may pose an ethical concern due to the harm it may cause; hence it will be unlikely for such a study to proceed, let alone succeed. A factor of compliance can also be questioned because coconut oil has a very distinct taste that may not be liked by many participants, which may affect the study. Before attempting to reach any conclusions, the long-term effects of coconut oil on LDL and, eventually, CVDs must be studied for several years. This is unlikely to be pursued for a variety of reasons, for example, the cost factor, the participation of a vast percentage of respondents who can remain compliant, and the influence of many other external factors such as dietary patterns, processed food intake, and the presence of other cardiovascular risk factors. Compliance with a specific dosage of coconut oil consumption over a long duration can be difficult if it is not part of their traditional/cultural diet. The studies also did not include the type of LDL particles involved, like large or small LDL particles, which seem to be more directly correlated than total LDL cholesterol. As a result, more regionally based research that examines the effects of various subtypes of coconut oil in dosages that vary from those employed in previous studies over a longer length of time seems to be the need of the hour. This could be done in areas/population that uses coconut oil as the main component of their daily cooking media.

Conclusion

After a systematic review of all the studies was done, it was found that even though coconut oil was popularly claimed as a healthy oil, there have not been enough human studies done to support that claim. Coconut oil substantially increased LDL and other lipid parameters, including HDL and overall total cholesterol, but the HDL ratio to total cholesterol still is not studied much. This increase in LDL was lower than butter and lard, but when compared to other tropical oils, mainly sunflower oil, some of the studies compared it with the effects on lipid parameters were not consistently different. Some studies claim that coconut oil had other effects such as improving antioxidant properties, reducing oxidant stress, and reducing cardiovascular health risk. However, we need to have more studies that combine oxidative properties and eventual cardiovascular risk to understand this. To understand anti-inflammatory properties, it takes to study a population over some time. Also, coconut oil increased HDL, which is called "good cholesterol," which was not the end focus of our study but could be influential in decreasing CVD propensity; the connection has not been explored extensively. Overall, data show that substituting coconut oil with cis-unsaturated fats reduces the risk of CVDs. As a result, this study refutes widespread assertions that coconut oil is a beneficial oil for lowering the risk of CVDs. Instead, this study shows that there need to be more studies done in this area from a functional and integrative standpoint with a more global approach by considering different types of coconut oil, different dosage patterns, diet patterns as a whole, other cardiovascular risk factors, and their influences.

## References

[1] (2021). Cardiovascular diseases. https://www.who.int/health-topics/cardiovascular-diseases.

[2] Chen Y, Freedman ND, Albert PS (2019). Association of cardiovascular disease with premature mortality in the United States. JAMA Cardiol.

[3] Vijayakumar M, Vasudevan DM, Sundaram KR (2016). A randomized study of coconut oil versus sunflower oil on cardiovascular risk factors in patients with stable coronary heart disease. Indian Heart J.

[4] Unhapipatpong C, Shantavasinkul PC, Kasemsup V (2021). Tropical oil consumption and cardiovascular disease: an umbrella review of systematic reviews and meta analyses. Nutrients.

[5] Schwingshackl L, Bogensberger B, Benčič A, Knüppel S, Boeing H, Hoffmann G (2018). Effects of oils and solid fats on blood lipids: a systematic review and network meta-analysis. J Lipid Res.

[6] Cabral CE, Klein MR (2017). Phytosterols in the treatment of hypercholesterolemia and prevention of cardiovascular diseases. Arq Bras Cardiol.

[7] Eyres L, Eyres MF, Chisholm A, Brown RC (2016). Coconut oil consumption and cardiovascular risk factors in humans. Nutr Rev.

[8] Neelakantan N, Seah JY, van Dam RM (2020). The effect of coconut oil consumption on cardiovascular risk factors: a systematic review and meta-analysis of clinical trials. Circulation.

[9] Elkhateeb YA, Alrashidi SB, Alshammary EH (2019). Assessment of knowledge about effects of coconut oil on human health among students of 
Hail University. Int J Biomed Sci.

[10] Khaw KT, Sharp SJ, Finikarides L (2018). Randomised trial of coconut oil, olive oil or butter on blood lipids and other cardiovascular risk factors in healthy men and women. BMJ Open.

[11] Júnior FA, Ruiz CR, de Oliveira Y (2021). Coconut oil supplementation does not affect blood pressure variability and oxidative stress: a placebo-controlled clinical study in stage-1 hypertensive patients. Nutrients.

[12] Palazhy S, Kamath P, Vasudevan DM (2018). Dietary fats and oxidative stress: a cross-sectional study among coronary artery disease subjects consuming coconut oil/sunflower oil. Indian J Clin Biochem.

[13] Pallazola VA, Davis DM, Whelton SP (2019). A clinician's guide to healthy eating for cardiovascular disease prevention. Mayo Clin Proc Innov Qual Outcomes.

[14] Niknamian S., Niknamian Niknamian, S. S. (2021). Dodecanoic-acid in extra virgin coconut oil, may reduce the incidence of heart disease and cancer in humans. Int J Sci Res.

[15] Saher F, Hosein M, Ahmed J (2018). Role of coconut oil pulling on oral health - an overview. J Pak Dent Assoc.

[16] Boateng L, Ansong R, Owusu WB, Steiner Asiedu M (2016). Coconut oil and palm oil's role in nutrition, health and national development: a review. Ghana Medical J.

[17] Dumancas G, Kasi Viswanath LC, de Leon AR (2016). Health Benefits of Virgin Coconut Oil. https://www.researchgate.net/publication/299371254_Health_benefits_of_virgin_coconut_oil.

[18] (2021). Saturated fat [internet]. https://www.heart.org/en/healthy-living/healthy-eating/eat-smart/fats/saturated-fats.

[19] Sacks FM, Lichtenstein AH, Wu JH (2017). Dietary fats and cardiovascular disease: a presidential advisory from the American Heart Association. Circulation.

[20] Glassner DL (2021). The association between type of fat and the risk of developing cardiovascular diseases. Sci J Lander College Arts Sci.

[21] Briggs MA, Petersen KS, Kris-Etherton PM (2017). Saturated fatty acids and cardiovascular disease: replacements for saturated fat to reduce cardiovascular risk. Healthcare (Basel).

[22] Jacobson TA, Maki KC, Orringer CE (2015). National lipid association recommendations for patient-centered management of dyslipidemia: Part 2. J Clin Lipidol.

[23] Sikand G, Severson T (2020). Top 10 dietary strategies for atherosclerotic cardiovascular risk reduction. Am J Prev Cardiol.

[24] Sacks FM (2020). Coconut oil and heart health: fact or fiction?. Circulation.

[25] Carro A, Panisello JM (2019). Deciphering the riddles in nutrition and cardiovascular disease. Eur Cardiol.

[26] Dayrit CS, Dayrit FM (2017). Coconut Oil: From Diet to Therapy. https://books.google.com/books?hl=en&lr=&id=1zSWDwAAQBAJ&oi=fnd&pg=PT3&dq=Coconut+Oil:+From+Diet+to+Therapy.+Quezon+City:+Anvil+Publishing,+Inc&ots=2HQ4lCYaRH&sig=YX8odk-QKsRUvCe-C8V5l8gfk9o#v=onepage&q&f=false.

